# Generation and analysis of large-scale expressed sequence tags (ESTs) from a full-length enriched cDNA library of porcine backfat tissue

**DOI:** 10.1186/1471-2164-7-36

**Published:** 2006-02-27

**Authors:** Tae-Hun Kim, Nam-Soon Kim, Dajeong Lim, Kyung-Tai Lee, Jung-Hwa Oh, Hye-Sook Park, Gil-Won Jang, Hyung-Yong Kim, Mina Jeon, Bong-Hwan Choi, Hae-Young Lee, HY Chung, Heebal Kim

**Affiliations:** 1 Division of Animal Genomics & Bioinformatics, National LivestockResearch Institute, Rural Development Administration, Omokchun-dong 564, Kwonsun-gu, Suwon, Korea; 2 Laboratory of Human Genomics, Genome Research Center, Korea Research Institute of Bioscience & Biotechnology, Daejeon, Korea; 3 School of Agricultural Biotechnology, Seoul National University San 56-1, Sillim-dong, Gwanak-gu, Seoul 151-742, Korea

## Abstract

**Background:**

Genome research in farm animals will expand our basic knowledge of the genetic control of complex traits, and the results will be applied in the livestock industry to improve meat quality and productivity, as well as to reduce the incidence of disease. A combination of quantitative trait locus mapping and microarray analysis is a useful approach to reduce the overall effort needed to identify genes associated with quantitative traits of interest.

**Results:**

We constructed a full-length enriched cDNA library from porcine backfat tissue. The estimated average size of the cDNA inserts was 1.7 kb, and the cDNA fullness ratio was 70%. In total, we deposited 16,110 high-quality sequences in the dbEST division of GenBank (accession numbers: DT319652-DT335761). For all the expressed sequence tags (ESTs), approximately 10.9 Mb of porcine sequence were generated with an average length of 674 bp per EST (range: 200–952 bp). Clustering and assembly of these ESTs resulted in a total of 5,008 unique sequences with 1,776 contigs (35.46%) and 3,232 singleton (65.54%) ESTs. From a total of 5,008 unique sequences, 3,154 (62.98%) were similar to other sequences, and 1,854 (37.02%) were identified as having no hit or low identity (<95%) and 60% coverage in The Institute for Genomic Research (TIGR) gene index of *Sus scrofa*. Gene ontology (GO) annotation of unique sequences showed that approximately 31.7, 32.3, and 30.8% were assigned molecular function, biological process, and cellular component GO terms, respectively. A total of 1,854 putative novel transcripts resulted after comparison and filtering with the TIGR SsGI; these included a large percentage of singletons (80.64%) and a small proportion of contigs (13.36%).

**Conclusion:**

The sequence data generated in this study will provide valuable information for studying expression profiles using EST-based microarrays and assist in the condensation of current pig TCs into clusters representing longer stretches of cDNA sequences. The isolation of genes expressed in backfat tissue is the first step toward a better understanding of backfat tissue on a genomic basis.

## Background

The breeding goals for pigs are largely directed towards retail carcass yield and meat quality because of the high economic value of these traits [1]. The marbling score, which is associated with the intramuscular fat (IMF) content, is one of the most important parameters for determining meat quality [2]. Backfat thickness (BFT) is moderately correlated with marbling traits [1]. BFT along with the average daily gain in mass have been the main selection traits among the finishing traits in the pig-breeding industry [1].

Quantitative trait locus (QTL) mapping and candidate gene analysis are currently being used to identify genes or markers associated with traits of economic interest. In the past decade, dozens of chromosome regions affecting traits related to fat deposition, such as IMF and BFT, in the pig have been reported using QTL mapping [51]. However, few of the genes controlling these QTL have been identified because a QTL may contain hundreds of potential polymorphic candidates.

The identification and localisation of genes expressed in tissues will enhance the selection and evaluation of candidate genes associated with QTL [3]. Expressed sequence tag (EST) projects provide a convenient and efficient approach for identifying and characterising the transcripts of genes expressed in tissues and cells. Moreover, the development of large-scale ESTs from various tissues has contributed to the construction of cDNA microarrays.

In pigs, the first EST project [4] and first large-scale EST project [5] have been reported. Subsequently, several research groups have generated ESTs from cDNA libraries constructed from various porcine tissues, such as anterior pituitary [6], backfat [7], brain [8], liver [9], skeletal muscle [10-12], orthopaedic implant-associated infection [13], and reproductive tissues [13-17].

Full-length cDNAs can be especially valuable resources for both functional genomics studies and the genomic structure of genes [18]. However, most porcine ESTs in public databases were derived from conventional cDNA libraries that have some drawbacks for isolating full-length cDNAs. In pigs, only two research groups have reported ESTs derived from full-length cDNA libraries constructed in the thymus, spleen, uterus, lung, liver, ovarian tissues, peripheral blood mononuclear cells [19], and olfactory bulbs [20]. However, ESTs generated from a full-length cDNA library constructed from backfat have not yet been deposited in a public database.

As a preliminary step towards developing large-scale EST sets expressed in adipose tissues and cells for the application of cDNA microarrays, we constructed a full-length enriched cDNA library from porcine backfat tissue using standard and normalised methods. In addition, we sequenced and characterised approximately 17,600 random clones.

## Results

### Characterisation of a porcine backfat cDNA library

To assess the quality of the full-length enriched cDNA library constructed from porcine backfat, the lengths and fullness ratios of cDNA inserts were investigated. As shown in Table 1, most of the cDNA insert sizes ranged from 1 to 3 kb based on 960 randomly selected clones from a non-normalised library. However, no cDNAs longer than 4 kb were found in these samples. The estimated average cDNA insert size of the library was 1.7 kb.

To evaluate the normalised library, redundancy rates were calculated in a clustering analysis of all ESTs generated from the non-normalised and normalised library using the program CAP3 [25]. The calculated redundancy rates of the non-normalised and normalised library were 72.08 and 14.48%, respectively. Although the redundancy rate of the normalised library was still high, it suggested that the library was successfully normalised.

In total, 300 sequences were randomly selected from the non-normalised library and compared to genes with e-values smaller than e^-100 ^in the nonredundant (nr) protein database of NCBI  using a BLASTX search to determine the fullness ratios of the library. Of these 300 sequences, 154 (51.3%) matched a known gene, and 70% of the clones of classified known genes were predicted to contain a putative ATG translation initiation codon.

### Virtual expression analysis of porcine backfat tissue

For an expression profile of backfat tissue, 1,331 contigs assembled from 13,991 high-quality ESTs from the non-normalised library were analysed. Of these, the 38 most abundant transcripts observed in this library are listed in Table 2. The contig with the largest number of ESTs was identified to be mitochondrial solute carrier family 25 member 6 (SLC25A6). Contigs with the second and third largest ESTs were eukaryotic translation elongation factor 1 gamma and actin (alpha skeletal muscle), respectively. Moreover, seven contigs of the 38 most abundant transcripts were related to the swine leukocyte antigen (SLA), especially SLA class I.

### Summary of the ESTs derived from a porcine backfat cDNA library

Our analysis considered 16,110 high-quality EST sequences (phred quality > 20 and at least 200 bp) consisting of 13,991 and 2,119 sequences derived from standard (15,360 clones) and normalised cDNA libraries (2,304 clones), respectively. All 16,110 sequences were deposited in the dbEST division of GenBank (accession numbers: DT319652-DT335761). For all ESTs, approximately 10.9 Mb of porcine sequence were generated with an average length of 674 bp per EST (range: 200–952 bp).

Clustering and assembly of these ESTs resulted in a total of 5,008 unique sequences with 1,776 contigs (35.46%) and 3,232 singleton (65.54%) ESTs. All unique sequences were compared to the protein database of GenBank using BLASTX and were functionally annotated as proposed by TIGR.

Of the 5,008 unique sequences, 3,154 (62.98%) were similar to other sequences, and 1,854 (37.02%) were identified as having no hit or low identity (<95%) and 60% coverage in the TIGR gene index of *Sus scrofa*.

### Gene Ontology annotation and bioinformatics analysis

Mainly from pig EST data, The Institute for Genomic Research (TIGR) has produced the Pig Gene Index (SsGI). The TIGR gene indices are based on clustering of EST sequences, and the elements of a cluster are evaluated to produce a set of unique, high-fidelity virtual transcripts, called Tentative Consensus (TC) sequences [29]. To date, TIGR has assembled pig ESTs with pig transcripts into 38,781 TC sequences.

**Figure 1:**
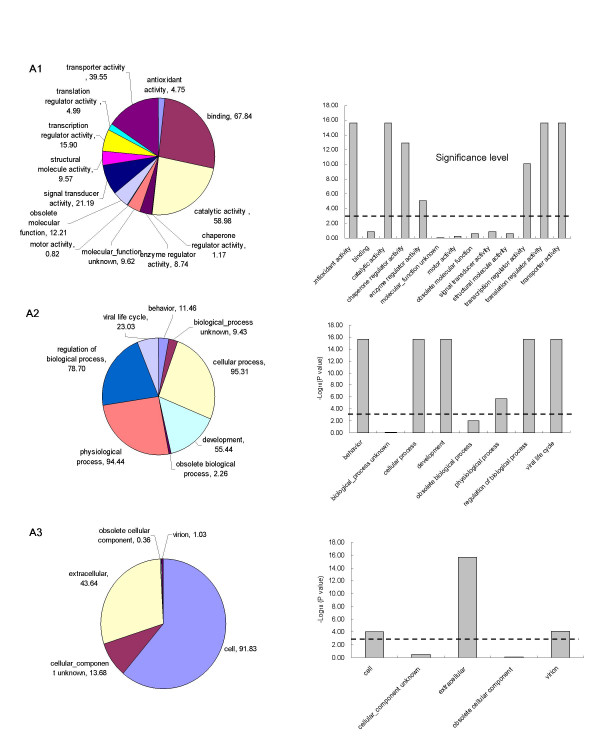
Gene Ontology annotation of the porcine backfat transcripts (A), andPearson's chi-square test of independence between the backfat transcripts and the TIGR SsGI (B). A1, A2 and A3 indicate molecular function, biological process, and cellular component, respectively. The overall significance level of alpharesulted from a Bonferroni correction, alpha = 0.5/m, where m = 30, as 30 GO terms were compared between backfat and the novel transcripts. The dotted line indicates the significance level.

In total, 5,008 unique sequences consisting of 1,776 contigs and 3,232 singleton ESTs were assigned the Gene Ontology (GO) terms using sequence comparisons with the TIGR SsGI. Subsets of the unique sequences were annotated with the GO terms. Of the unique sequences, 31.7, 32.3, and 30.8% were assigned to molecular function, biological process, and cellular component GO terms, respectively. These are not mutually exclusive terms. Figure 1A shows the 2^nd^-level GO term distribution of the unique sequences. We compared the 2^nd^-level GO terms annotation of the TIGR SsGI and our unique sequences using Pearson's chi-square test (Figure 1B). Most of the terms in which the *p*-value showed significance were enriched in our unique sequences. This indicates that the normalisation and identification of new functional genes from our full-length cDNAs were very efficient. For example, chaperone regulator activity, the smallest child term (0.03%) of molecular function in the TIGR SsGI, was enriched 39-fold (1.17%) in our sequences.

A total of 1,854 putative novel transcripts resulted after the comparison and filtering with the TIGR SsGI. These included a large proportion of singletons (80.64%) and a small percentage of contigs (13.36%).

Of the 1,854 ESTs including 359 contigs and 1,495 singletons that had no hit or low identity (<95%) at the TIGR gene index of *S. scrofa*, 48.98% (908) had no significant hits to the nonredundant (nr) protein databases of the NCBI using a BLASTX search (e-value < 0.00001). As expected, this implied that most of the singletons with no BLAST hit are not genuine protein-coding sequences.

## Discussion

Genome research in farm animals will expand our basic knowledge of the genetic control of complex traits, and the results will be applied in the livestock industry to improve meat quality and productivity, as well as to reduce the incidence of disease [30]. A combination of quantitative trait locus mapping and microarray analysis is a useful approach to reduce the overall effort needed to identify genes associated with quantitative traits of interest [31].

This is the first paper related to the construction and large-scale EST analysis of a full-length enriched cDNA library from porcine backfat tissue. The estimated average cDNA insert size of this library was 1.7 kb, which was no smaller than for other full-length cDNA libraries [20,32]. In addition, the fullness ratio was 70%, which was similar to 60–70% for full-length enriched cDNA libraries constructed using methods of selecting the cap structure [21,22,33-37]. For the normalised library, the redundancy rate was reduced by approximately five times when the library was normalised in this study. Our result was similar to a reported reduction of 4.3 times [8]. The redundancy rate of ESTs from the non-normalised library was extremely high compared with those of the other cDNA libraries that have been constructed using our method (data not shown). The redundancy rate of the non-normalised library may still be high owing to the biological characteristics of porcine backfat tissue. As shown in Table 2, the 38 most abundant transcripts include 35.5% of the 13,991 ESTs generated from the non-normalised library. The library described was successfully normalised and proved to be an excellent resource for collecting unique transcripts expressed in porcine backfat tissue. In addition, these inserts were ligated unidirectionally into a pCNS-D2 plasmid vector [23] that can be expressed in mammalian cells. These clones could be readily used for expression and function studies, and will prove a valuable resource for functional genomics, as well as for genome annotation.

In this study, 16,110 high-quality ESTs were generated from 17,664 random clones and deposited into GenBank. To analyse the 16,110 ESTs efficiently, they were clustered into putative unique transcripts, and GO analysis was performed for functional studies. Sequences expressed in porcine backfat tissue contained a high percentage of chaperone regulator activity (39 times), antioxidant activity (4.9 times), translation regulator activity (3.7 times), and transporter activity (2.6 times) compared to total pig TCs in a molecular function category. Strangely, the GO terms of the viron and viral life cycle were significantly enriched in the backfat library compared with the SsGI. Backfat tissue consists of several different kinds of cell types and some studies have shown severe macrophage infiltration of obese adipose tissue [49,50]. Currently, we are reluctant to make hasty biological inferences from the data without validating it experimentally. Therefore, further study is needed to elucidate why the GO terms of the viron and viral life cycle are enriched in the backfat library. This suggests that ESTs deposited in the TIGR database were generated from various kinds of tissues or growth/developmental stages of a certain tissue or germinal cells. The percentage of sequences within different categories of the gene ontology index showed different distributions according to tissues used for the construction of the cDNA library [12,17,20,38,39]. Although our data are limited by the fact that this library was constructed from pooled samples collected at various growth stages, a functional categorisation of ESTs with known gene matches underlines general differences in the expression profiles of different tissues.

To identify novel sequences, the sequences of SsGI (ver 11.0) and our unique sequences were compared using the stand-alone BLAST program (ver 2.29) of NCBI. As a result, we obtained 1,854 putative novel transcripts with no BLAST hits. Novel transcripts may be expressed differently among tissues. In particular, we suggest that many of the novel transcripts observed in this study are expressed only in backfat tissue. However, we suggest that a large number of novel transcripts resulted from the lack of ESTs derived from porcine backfat tissue in the TIGR database. Moreover, several porcine ESTs in the TIGR SsGI were generated based on 3' end sequencing, while our sequences were produced from the 5' end. Sequence information for a certain EST in the TIGR database may show insufficient overlap between our sequences and sequences in the TIGR database to identify high similarity, although the two sequences may actually represent the same gene.

To date, approximately 400,000 ESTs derived from porcine tissues have been deposited in public databases. TIGR released SsGI ver 11.0 (18 January 2005), which consists of 38,781 TC sequences, 65,000 singleton ESTs, and 546 singleton ETs generated from approximately 400,000 ESTs. However, relatively few ESTs derived from porcine backfat tissue are stored in public databases. Before our study, 298 ESTs from porcine backfat tissue were assigned to a porcine radiation hybrid map [7]. Only 49 ESTs generated from a cDNA library from porcine backfat tissue and 54 ESTs derived from a cDNA library constructed from porcine *longissimus dorsi *muscle and backfat tissue have been deposited in GenBank. By contrast, approximately 130,000 ESTs generated from a full-length cDNA library are available from GenBank, and the Pig EST data explorer (PEDE) provides 68,076 ESTs consisting of 5,546 contigs and 28,461 singletons from full-length cDNA libraries constructed from various tissues [19]. However, ESTs derived from a full-length cDNA library constructed from porcine backfat tissue have not yet been deposited in public databases.

The most abundant transcript in porcine backfat tissue, which represented 4.9% of the ESTs derived from the non-normalised library, was the mitochondrial solute carrier family 25 member 6, which plays a fundamental role in cellular energy metabolism (Table 2). The most abundant transcripts expressed in backfat tissue were also expressed in olfactory bulbs of 5-week-old pigs [20]. Mitochondrial solute carrier family 25 member 6 was also the most abundant transcript in the olfactory bulbs of 5-week-old pigs. The expression of this gene has been reported to decrease when the cells are induced to differentiate in humans [40]. The most abundant genes were related to proteins involved in cell structure, cell growth, and basic cell functions. Elongation factor 1, which is essential for protein synthesis, was the fourth most abundant transcript. It was expressed secondarily in porcine Peyer's patch [39]. Seven of the 38 most abundant genes were related to SLA. The nomenclature for factors of the SLA class-I system [48] classifies them into four alleles (SLA-1*w13ms21, SLA-1*w08sz01, SLA-1*w11yn01, SLA-1*w09ms09) in class-1 group, two alleles (SLA-2*05sy01, SLA-2*w09sn01) in class-2 group, and one allele (SLA-3*0101) in class-3 group of SLA class I. Of these, SLA-1*w13ms21 is the most abundantly expressed SLA allele in backfat tissue. The swine leukocyte antigen is also the most abundantly expressed transcript in oocytes during embryogenesis in pigs [17]. Vimentin, which encodes an intermediate filament protein, was the tenth most abundant transcript in our study. The frequency of sequences obtained for a particular gene is proportional to the level of expression of that gene because all sequences were obtained through random selection of clones [42,43]. By contrast, a total of 1,651 unique sequences with 270 contigs and 1,651 singlets were generated from 2,269 high-quality sequences from 2,302 clones of the normalised library. Thirty-eight of the 270 contigs assembled three or more sequences, of which the most abundant transcript composed of 8 ESTs was matched with haplotype H01 of SLA class I (AJ131112). Moreover, 9 of 38 contigs were not observed in sequences generated from the non-normalised library, of which five contigs were putative noble transcripts and the others were matched with ITBA1 protein (XM_864356), FATZ related protein 3 (AJ300587), bone morphogenetic protein 7 precursor (NM_001719), and growth factor (NM_005262). However, we did not find backfat-specific ESTs among the abundantly expressed transcripts in the normalised library.

DNA microarray technology is a useful tool for studying the expression of a large number of genes in a particular tissue or cell type and allows an efficient, objective, and quantitative evaluation of genes in the QTL. It has the potential to reduce the overall effort needed in identifying genes causally associated with quantitative traits of interest [31,44].

To date, porcine cDNA microarrays have been reported using EST sets derived from skeletal muscle [45], whole embryo and adult skeletal muscle, and differential display PCR products from foetal and postnatal muscle [41], brain [8], small intestine [46], and ovarian follicle [47]. More recently, oligonucleotide arrays, such as QIAGEN Array-Ready Oligo Set for the Pig Genome (version 1.0), the Pig Genome Oligo Extension Set (version 1.0), and the Affymetix GeneChip Porcine Genome Array, representing 20,201 genes, have been made available commercially. However, large-scale EST data sets are essential resources for constructing comprehensive cDNA or oligonucleotide arrays. Therefore, the EST information generated in this study will be valuable for studying expression profiles using EST-based microarrays and will assist in the condensation of current pig TCs into clusters representing longer stretches of cDNA sequences.

In conclusion, approximately 70% of the full-length enriched cDNA library of porcine backfat tissue constructed in this study consisted of full-length cDNAs. Moreover, because pCNS-D2, the plasmid vector used for this library, can be expressed in mammalian cells, these clones could be used readily for expression and function studies. These sequence data will provide valuable information for identifying porcine genes expressed in backfat tissue. The isolation of genes expressed in backfat tissue is the first step towards a better understanding of backfat tissue on a genomic basis. The nonredundant ESTs generated from this library will allow us to construct cDNA microarrays for expression assays and to localise them onto a physical map for assessing genes that regulate fat accumulation in backfat tissue and further affect meat quality in pigs. Moreover, these expressed sequences will contribute towards expanding the comparative map between the pig and mammals such as humans and mice that have well characterised genomes.

## Methods

### Sample preparation and total RNA extraction

Four Landrace × Large White crossbred pigs were slaughtered at 1, 7, 12, 18, and 24 weeks of age to maximise the discovery of backfat expressed genes, as well as to identify nucleotide sequence variation for future studies. The backfat was excised immediately after slaughtering, snap frozen in liquid nitrogen, and stored at -70°C until RNA was extracted. Total RNA was extracted from all the samples using an RNeasy Lipid Tissue Midi Kit (Qiagen, Valencia, CA, USA) as per the manufacturer's instructions.

### Full-length enriched cDNA library construction

To construct a full-length enriched cDNA library, the prepared total RNA was pooled with the same amount of each sample. A full-length enriched cDNA library was constructed as previously described [21]. Briefly, 100 μg of total RNA were treated with bacterial alkaline phosphatase (TaKaRa, Tokyo, Japan) and then with 100 units of tobacco acid pyrophosphatase (Waco, Tokyo, Japan). The pretreated total RNA was ligated with 0.4 μg of 5'-ligoribonucleotide (5'-AGC AUC GAG UCG GCC UUG UUG GCC UAC UGG-3'). After completing the oligo capping reactions, mRNA was isolated using an Oligotex Mini Kit (Qiagen). The synthesis of first-strand cDNA from the purified mRNA and the cDNA amplification were performed as previously described [22]. The amplified PCR products were then digested with *Sfi*I, and cDNAs longer than 1.3 kb were ligated into *Dra*III-digested pCNS-D2 [23] in an orientation-defined manner. The ligated cDNA was then transformed into *Escherichia coli *Top 10F' (Invitrogen, Carlsbad, CA, USA) by electroporation (Gene Pulser II; BioRad, Hercules, CA, USA). The constructed cDNA library was normalised as previously described [24]. Consequently, two libraries were constructed from the same porcine backfat sample: a full-length cDNA library and a normalised full-length cDNA library.

### Plasmid isolation and cDNA sequencing

Colonies were picked randomly, inoculated into individual wells of 96-deep well plates containing 1 mL of LB media and incubated at 37°C for 18 h. Plasmid DNAs were extracted using a Montage Plasmid Miniprep 96 Kit (Millipore, Billerica, MA, USA) according to the manufacturer's instructions. The cDNA inserts were sequenced once from the 5' end of clones using a BigDye Terminator Sequencing Kit ver 3.1 (Applied Biosystems, Foster City, CA, USA) and a 3730 DNA Analyser (Applied Biosystems).

### Characterisation of the full-length enriched cDNA library

To evaluate the quality of the full-length enriched cDNA library constructed in this study, the lengths and fullness ratios of the cDNA inserts were investigated. In total, 960 clones were selected from the library randomly, and plasmid DNAs were extracted and digested with *Eco*RI and *Not*I restriction enzymes. Size fractionation was performed in a 1% agarose gel containing ethidium bromide.

We selected 300 sequences to determine the fullness ratios of the library. The sequences were compared to the nonredundant (nr) protein database at the National Center for Biotechnology Information (NCBI; ) using a BLASTX search. Sequences matching the genes with an e-value smaller than e^-100 ^were selected, and then we evaluated whether a putative translation start site existed in these sequences. When a sequence contained a putative translation initiation codon, the sequence was defined as a full-length cDNA.

### Sequence data analysis and EST clustering

The porcine EST trace data were base-called using the program phrep [25] from trace chromatogram data of 5' EST sequences. The EST reads were quality trimmed using the phred quality score at a position where five ambiguous bases (phred quality > 2 and at least 200 bp) were found within 15 consecutive bases. The vector sequences of the base-called EST data were clipped. The porcine EST data described here have been submitted to the GenBank database. The EST sequences were clustered and assembled using the cross-match program and the program CAP3 [26].

### Gene Ontology annotation and bioinformatics analysis

To classify our ESTs and unique sequences using Gene Ontology (GO) terms, we downloaded The Institute for Genomic Research (TIGR) Pig Gene Index (SsGI; Release 11.0). A sequence similarity comparison between the Tentative Consensus (TC) sequences of the SsGI and our sequences was performed using the stand-alone BLAST program of NCBI (version 2.2.9), with 95% identity and a 500-bit score as the cutoff values. The unique sequences were categorised using terms from the GO database. Since a large portion of these pig sequences has not yet been annotated, gene annotation was performed by extracting information with the term already annotated. Pearson's chi-square test was used to test the significance of which GO terms were enriched in one data set, but relatively depleted in the other. We compared the 2^nd^-level GO terms of the TIGR SsGI and our unique sequences using Pearson's chi-square test. As described previously [27], a particular GO term can be viewed as a function that maps gene G in go (G) = 0 or 1, according to the corresponding GO term. The null hypothesis of no association between gene lists and a particular GO term is translated into equal distributions of binary random variables. Only the list of genes annotated with GO terms was counted for the test. A Bonferroni correction [28] was applied to correct the multiple test problems.

To identify putative novel transcripts, SsGI sequences (Release 11.0) and our unique sequences were compared using the stand-alone BLAST program. Sequences with no BLAST hits represented putative novel transcripts. Some of the sequences were discarded if they contained fewer than 200 bp.

## Authors' contributions

THK conceived of the study, participated in the coordination of bioinformatics activities and data analysis, and drafted the manuscript. NSK and JHO constructed the full-length enriched library. GWJ, BHC, and HYC participated in the sequence processing and library evaluation. KTL, HSP and HYL performed the high-throughput EST sequencing and quality assurance. DL, MJ, HYK, and HK performed the EST assembly, clustering, and annotation.

## Figures and Tables

**Table 1:** Size distributions of the selected cDNA inserts.

Size (kb)	Number of clones*	Frequency (%)
No insert	45	4.7
< 0.5	45	4.7
0.5 ~ 1.0	67	7.0
1.0 ~ 1.6	441	45.9
1.6 ~ 2.0	298	31.0
2.0 ~ 3.0	107	11.2
3.0 ~ 4.0	2	0.2
Total	960	100.0

*The clones were selected from the non-normalised library.

**Table 2:** Results of the BLAST search for contigs composed of the 38 most redundant ESTs in the non-normalised library from porcine backfat tissue

Contig (bp)	No of ESTs	Accession no	Species	NCBI Blast	
				
				Gene name	e-value
contig375 (983)	685	NM_214418	Pig	Mitochondrial solute carrier family 25 member 6	0.0
contig1036 (940)	350	NM_001404	Human	Eukaryotic translation elongation factor 1 gamma	0.0
contig54 ('1,646)	327	NM_174225.1	Cattle	Actin, alpha skeletal muscle	0.0
contig1727 (1,776)	312	AF034253	Pig	Sus scrofa mitochondrion, complete genome	0.0
Contig1227 (1,442)	293	XM_845314	Dog	Eukaryotic translation elongation factor 1 alpha 1	0.0
contig265 (950)	276	AY459297.1	Pig	Sus scrofa MHC class I antigen (SLA-1), SLA-1*ms21 allele	0.0
contig155 (1,589)	241	NM_005165	Human	Aldolase C, fructose-bisphosphate	0.0
contig1336 (984)	229	NM_001014894.1	Cattle	Ribosomal protein L4	0.0
contig34 (880)	203	NM_015710.3	Human	Glioma tumor suppressor candidate region gene 2 protein (p60)	0.0
contig1237 (1,230)	178	NM_002593.2	Human	Procollagen C-endopeptidase enhancer	0.0
contig1086 (1,358)	168	NM_003380.2	Human	Vimentin	0.0
contig944 (1,173)	124	NM_174049.1	Cattle	Enolase 1	0.0
contig1115 (964)	112	NM_001005726.1	Pig	Annexin A2	0.0
contig324 (1,173)	112	AF464013.1	Pig	Sus scrofa MHC class I antigen (SLA-1), SLA-1*z allele	0.0
contig1661 (913)	104	AY102469.1	Pig	Sus scrofa MHC class I antigen (SLA-P1), SLA-P1*yn allele	0.0
contig1394 (899)	85	AY135594.1	Pig	Sus scrofa MHC class I antigen (SLA-1), SLA-1*mel9 allele	0.0
contig661 (1,167)	81	NM_006082.2	Human	Tubulin alpha-1 chain	0.0
contig1090 (1,141)	76	NM_213920.1	Pig	Decorin	0.0
contig502 (1,000)	72	NM_006088.5	Human	Tubulin beta-2	0.0
contig1688 (881)	69	XM_614355	Cattle	Bos taurus similar to heat shock protein, alpha-crystallin-related	0.0
contig542 (1,167)	62	NM_001154.2	Human	Annexin A5	0.0
contig1269 (873)	57	AF464005.1	Pig	Sus scrofa MHC class I antigen (SLA-B), SLA-B*y allele	0.0
contig671 (876)	56	NM_182810.1	Human	Cyclic-AMP-dependent transcription factor ATF-4	0.0
contig1601 (1,220)	55	NM_174180.2	Cattle	Short-chain dehydrogenase/reductase 1	0.0
contig715 (891)	52	NM_004925.3	Human	Aquaporin 3	0.0
contig130 (873)	49	AF464059.2	Pig	Sus scrofa MHC class I antigen (SLA-B), SLA-B*n allele	0.0
Contig284 (951)	49	NM_001035344	Cattle	Bos taurus Mof4 family associated protein 1 (MRFAP1),	0.0
contig192 (897)	48	NM_001013585.2	Cattle	Endothelial differentiation, sphingolipid G-protein-coupled receptor, 1	0.0
contig848 (863)	48	NM_174715.1	Cattle	Ribosomal protein L3	0.0
contig549 (898)	47	NM_214276.1	Pig	Citrate synthase	0.0
contig861 (898)	47	XM_587033	Cattle	Angiopoietin-like 4	8e-109
contig884 (896)	47	NM_001823.3	Human	Creatine kinase, brain	0.0
contig638 (1,944)	45	NM_214246.1	Pig	Carboxylesterase	0.0
contig657 (1,792)	44	NM_023948.3	Human	Motile sperm domain containing 3	0.0
Contig1616 (958)	44	Q9XSD9	Pig	PGS2_PIG (Q9XSD9) Decorin precursor (Bone proteoglycan II) (PG-S2)	0.0
contig939 (876)	41	AJ131112	Pig	Sus scrofa MHC class I SLA genes, haplotype H01	5e-119
contig33 (1,008)	40	NM_213774.1	Pig	CD74 antigen	0.0
contig817 (2,268)	40	NM_006472.1	Human	Thioredoxin interacting protein	0.0
